# How Can Students Feel More Vital Amidst Severe Restrictions? Psychological Needs Satisfaction, Motivational Regulation and Vitality of Students during the Coronavirus Pandemic Restrictions

**DOI:** 10.3390/ejihpe11020030

**Published:** 2021-05-08

**Authors:** Daniela Martinek, Matteo Carmignola, Florian H. Müller, Sonja Bieg, Almut Thomas, Alexander Eckes, Nadine Großmann, Ann-Kathrin Dittrich, Matthias Wilde

**Affiliations:** 1 School of Education, University of Salzburg, Erzabt-Klotz-Str.1, 5020 Salzburg, Austria; matteo.carmignola@sbg.ac.at; 2 Institute of Instructional and School Development, University of Klagenfurt, Sterneckstraße 15, 9020 Klagenfurt am Wörthersee, Austria; florian.mueller@aau.at; 3 Department of Psychology, University of Education of Weingarten, Kirchplatz 2, 88250 Weingarten, Germany; sonja.bieg@ph-weingarten.de; 4 Department of Primary Education, University College of Teacher Education Carinthia, Hubertusstraße 1, 9020 Klagenfurt am Wörthersee, Austria; almut.thomas@ph-kaernten.ac.at; 5 Department of Biology Didactics, Osnabrück University, Barbarastr. 11, 49076 Osnabrück, Germany; alexander.eckes@uos.de; 6 Department of Biology Didactics, Bielefeld University, Universitätsstraße 25, 33615 Bielefeld, Germany; nadine.grossmann@uni-bielefeld.de (N.G.); matthias.wilde@uni-bielefeld.de (M.W.); 7 Department for Teacher Education and School Research, University of Innsbruck, Fürstenweg 176, 6020 Innsbruck, Austria; Ann-Kathrin.Dittrich@uibk.ac.at

**Keywords:** Self-Determination Theory, distance learning, psychological needs satisfaction and frustration, higher education, motivation, vitality

## Abstract

During the pandemic restrictions imposed in spring 2020, many aspects of students’ living and learning environments changed drastically. From the perspective of Self-Determination Theory, changes in social context interact with the satisfaction or frustration of basic psychological needs and, as a result, with study-related motivational regulation and vitality. In this study, we investigate the relationships between the contextual factors of online-based distance learning, basic psychological needs, forms of motivational regulation and subjective vitality in a sample of *N* = 1849 university students across eight universities in Austria and Germany. Based on structural equational modelling, the results stress the relevance of satisfaction with technological resources in regard to higher levels of satisfaction in all three basic psychological needs, while perceived overload is linked to lower levels of needs satisfaction and increased basic psychological needs frustration. Further, the estimated workload difference before and during the pandemic is not related to the motivational outcomes of the model. All relationships have been tested for mediation effects between basic psychological needs and the different forms of motivational regulation on subjective vitality: for the need for relatedness, no mediation is found, while the effect of the need for autonomy is fully mediated by autonomous regulation styles. The need for competence was associated with several mediating interactions with regulation styles. The results offer insight into students’ perceptions of their study-related experiences during the pandemic and can help to develop effective methods in online-based and blended learning settings in the future.

## 1. Introduction

Due to restrictions imposed in spring 2020 during the pandemic, students’ lives changed drastically with little prior notice. Besides severe restrictions in their everyday lives, students in higher education had to switch from studying on-site to distance learning. This sudden switch led to changes in workload, inadequate assignments, or a lack of information. Furthermore, problems with the technical equipment hampered students’ learning and motivation [1]. Moreover, students reported that they miss personal contact with their fellow students when constrained to online learning [1,2] (Krammer et al., 2020; Wang et al., 2019; Wong, 2020).

This substantial change in students’ social context which, according to Self-Determination Theory [3] influences the inner organisation and integration of the self, is likely to have affected students’ satisfaction within three basic psychological needs (BPN): autonomy, competence and relatedness, as well as their motivational regulation and vitality. Students’ basic psychological needs satisfaction or frustration is most likely affected by contextual conditions during distance learning, such as the availability and quality of technical equipment, type of course, the quality of interaction between students and teachers or changes in workload. Such contextual conditions can vary widely and be perceived differently by students. Therefore, this paper examines the contextual conditions that may have contributed to the satisfaction or frustration of students’ basic psychological needs during the pandemic restrictions, and their association with students’ motivational regulation and vitality.

## 2. Theoretical Background Based on Self-Determination Theory

### 2.1. Satisfaction of Students’ Basic Psychological Needs

Within SDT, the three basic psychological needs of autonomy, competence and relatedness are seen as innate and universal, and needs satisfaction significantly contributes to organismic growth and psychological well-being in terms of full psychological functioning [4,5]. The satisfaction of students’ psychological needs is affected by the study environment, which may be more or less supportive, or even suppressive, as was possibly the case due to the pandemic restrictions [6]. Students’ experience autonomy when they perceive themselves to be the originators of their behaviour and when studying it allows them to follow their interests and personal values [7]. At university, students’ psychological need for autonomy can be satisfied by the provision of meaningful choices and transparent rationales for requested behaviour, as well as opportunities to work in a self-determined manner [8]. Notably, autonomy has to be distinguished from individual freedom, which was severely reduced during the restrictions because the former can be experienced whenever a behaviour accords with integrated values. That is why, even with severe external restrictions, such as those imposed by politicians or university lecturers during the first peak of the pandemic, students’ need for autonomy can be satisfied, provided that they are able to understand the rationale for the requested behaviour and to identify with the intended goals and consequences [9]. To what extent this is the case is one of the questions to be investigated in this study.

Students’ need for competence is satisfied if they can explore their skills and capabilities successfully and feel effective in their interactions with social environments [10]. Feeling able to master study-related tasks and experiencing an individual sense of self-efficacy in action are hallmarks of the satisfaction of the need for competence. Due to the pandemic restrictions, students’ interactions in the social environment and opportunities to experience competence when studying were altered: during this period, students were not allowed to attend university or meet colleagues or lecturers, and face-to-face lectures were replaced with virtual formats.

Students’ need for relatedness is satisfied if they have the opportunity to care for others and are cared for by significant others [8,11]. The need for relatedness is expressed by the willingness to share personal resources with others and the development of a sense of belonging and security [12,13]. Due to the restrictions, students’ need for relatedness, as well as their need for competence, may have been thwarted, as personal contacts had to be limited and students were forced to rely on digital technology to communicate with their colleagues and lecturers. In a recent study, Wong [14] was able to show that social integration when studying online, in particular, was significantly lower during the pandemic restrictions. Given this finding, it seems to be much more difficult to ensure relatedness to lecturers and fellow students when engaged in distance learning than it is face-to-face.

Recently, needs frustration, which is more than a lack of needs satisfaction, has attracted increased attention within SDT because it further enhances our understanding of motivational processes [15]. Whereas needs dissatisfaction is the result of a study environment which is indifferent to needs satisfaction [2], needs frustration occurs in contexts that are experienced as needs-thwarting [6]. Students experience need frustration if their needs are actively suppressed (e.g., by lecturers or peers) and they feel pressured to think, feel or behave in an expected way. Thus, it is quite conceivable that the needs are not frustrated in times of the pandemic, but at the same time students’ needs cannot be decisively satisfied. That is, need frustration and need satisfaction are not two sides of the same coin. Overall, it should be noted that high satisfaction and low frustration of basic psychological needs are both relevant for physiological and psychological well-being, ideal development, mental health, autonomous regulation and the perception of subjective vitality [16].

### 2.2. Motivational Regulation

According to Deci and Ryan, intrinsically motivated behaviour represents the prototype of self-determined behaviours and, as such, can be described as ‘wholly volitional, as representative of and emanating from one’s sense of self’ [17] (p. 5). Intrinsic motivation exists alongside feelings of joy, curiosity, exploration, spontaneity and interest. It is considered a manifestation of learning and development and constitutes a source of vitality [10]. In addition, SDT assumes four types of extrinsic motivation, referred to as regulation styles. All extrinsically motivated behaviours aim at an end state that is separate from the actual behaviour. The four regulation styles vary in their degree of self-determination, their locus of causality and the level to which they integrate values and norms into the ‘autonomous self’ [3]; motivational regulation is sensitive to changes in the environment, such as those caused by the pandemic.

Types of regulation: (1) External regulation is the least autonomous form and characterises behaviours that are conducted to satisfy an external demand, such as to obtain rewards or to avoid punishment or control [10]. Studies conducted on university students show that external regulation is associated with negative affect, low life satisfaction [18] and low levels of well-being [19]. (2) Introjected regulation is partly internalised to the self and occurs in behaviours to comply with poorly integrated social norms. It is associated with either ego-enhancement or the avoidance of ego-depletion [10] and thus contains positive aspects (e.g., pride) as well as negative ones (e.g., shame) [20]. Originally, introjected regulation was conceptualised as a one-dimensional construct but, due to inconsistent associations with various outcomes [21], a distinction was needed between the approach and avoidance components [22,23]. Recent studies on university and secondary-school students show that introjected approach regulation is positively associated with well-being, whereas introjected avoidance regulation is negatively associated with well-being and positively associated with negative affect [22,23]. (3) Identified regulation is characterised by a high degree of perceived autonomy pertaining to personally important goals [4]. With identified regulation, actions are guided by an understanding of and identification with the value of actions taken [22]. Actions are considered self-determined and are associated with many beneficial outcomes, such as a negative association with negative affect and a positive association with life satisfaction and perceived autonomy support [18]. A student may not be particularly interested in a certain subject but, when graduation is of personal relevance, their learning regulation will be deemed to be identified. (4) Integrated regulation comprises behaviours that are entirely congruent with internalised values, so that they coexist harmoniously with other aspects of the self [17]. Integrated regulation shares qualities with intrinsic motivation [24] and it is empirically difficult to differentiate between them [25]. Hence, in our empirical study, this regulatory style has not been considered.

Well-integrated regulations, such as the intrinsic and identified models, build on experiences of choice and volition and are, therefore, denoted as autonomous regulation, whereas introjected and external regulations derive from external demands and are deemed to be controlled regulation [24,26]. Studies show that controlled regulation is associated with negative outcomes [3] such as increased drop-out rates [27], whereas autonomous regulation is associated with positive learning outcomes [26], enhanced subjective well-being and vitality [10], not only in university students [3,27].

### 2.3. Vitality

According to Ryan and Deci [3,11], vitality is the energy available to the self and is a salient and functionally significant indicator of health and motivation. Vitality is the experience of mental and physical energy, enthusiasm and a general thirst for life [28]. Students feel vital if they are functioning effectively ‘in sync’ with their inner self [4]. Vitality is facilitated by high levels of psychological needs satisfaction paired with low levels of needs frustration [29,30]. Vitality, which follows diurnal cycles, is influenced by somatic factors, such as exercise, rest and nutrition, and by psychological factors, such as students’ energy for controlling their behaviours and suppressing impulses, and their motivational regulation [7]. Using the ego-depletion model, Baumeister, Muraven and Tice [31] suggest that autonomous activities do not deplete energy, whereas activities with an external locus of causality do, resulting in lower vitality. As such, the more students perceive an external locus of causality for studying, the lower their vitality will be. This can be applied to students’ regulation types in relation to studying during the pandemic restrictions, with controlled regulation showing the strongest negative effect on vitality, and autonomous regulation showing the strongest positive impact. In former studies, positive affect [32], greater stimulation and productivity, better stress management and mental health, and greater resilience to physical and viral stressors [33,34] have been associated with subjective vitality.

### 2.4. Mandatory Distance Studying

Distance learning comes with several challenges [2,14,35]. In particular, the lack of technical equipment and the technical availability of online-learning environments have been identified as pivotal predictors for users’ frustration with distance learning [36]. Moreover, workload can predict (dis-)satisfaction or frustration with online learning. It is, however, hard to evaluate the importance of workload, as previous research on online-based learning [37,38] does not sufficiently differentiate between the subjective burden of perceived overload and the actual workload (time spent in studying activities), as suggested by SDT [3]. Furthermore, experimental research suggests considering the interaction between workload and motivational regulation to investigate its specific impact on emotional and motivational outcomes [39].

The short-term switch to distance studying during the first lockdown in spring 2020 affected several characteristics of teaching and learning in higher education, such as workload, temporal burden, study requirements, and social relations [1,40], all of which may have an impact on students’ basic psychological needs satisfaction and needs frustration and subsequently on their vitality and learning motivation. Insufficient teacher feedback, high workload, and insufficient technical equipment may, for instance, undermine students’ competence satisfaction. Indeed, empirical evidence suggests that during the COVID-19 pandemic competence satisfaction became the strongest predictor of university students’ well-being [40]. At the same time, full distance learning provided students with greater latitude for time management [1] and thus, may have supported autonomy satisfaction. Holzer et al. [40] found only moderate associations between university students’ autonomy satisfaction and intrinsic learning motivation during the pandemic. Evidently, the obligation for distance learning also affected the communication with peers. Research indicates that limitations in social contact during the COVID-19 pandemic predict students’ vitality and well-being [41]. In contrast to studies before the pandemic [42], the association between social relatedness and well-being or life satisfaction was only small [40,41].

## 3. The Current Study

In this study, we not only analysed the psychological needs of students during the pandemic restrictions, bearing in mind the organismic dialectical approach of SDT, but also integrated selected contextual factors which may have an important influence on distance learning. In detail, we investigated whether satisfaction with technology, estimated differences in workload associated with the restrictions, and perceived overload while studying during the pandemic restrictions were related to the satisfaction or frustration of basic psychological needs and, mediated through autonomous and controlled forms of regulation, the perceived vitality of students.

Following a correlative design, the study tested the following hypotheses:

- **Hypothesis 1 (H1).** The assessment of satisfaction with technology reports positive relations with the satisfaction of all basic psychological needs [2], while perceived overload and the difference in weekly workload should be associated with lower levels of needs satisfaction and higher frustration of basic psychological needs [38].
- **Hypothesis 2 (H2).** The satisfaction of all three basic psychological needs is associated with more autonomous forms of regulation [5], while controlled regulation is linked to higher levels of need frustration [15].
- **Hypothesis 3a (H3a).** Subjective vitality is predicted by basic psychological needs satisfaction and frustration [29], as well as by motivational regulation [11]. Here, we expect a positive relation between needs satisfaction and autonomous regulation [43], and that needs frustration will be positively related to controlled regulation [6].
- **Hypothesis 3b (H3b).** The relation between basic psychological needs and vitality will be mediated [44] by motivational regulation types.

## 4. Methods

### 4.1. Participants

A total sample of 1849 students from eight universities in Austria and Germany participated in this online-based survey in a time window of three weeks starting from mid-May 2020, which marks the easing of the most severe restrictions of the first pandemic wave in Central Europe. Participation was voluntary and students were contacted via the e-mail accounts linked to their institutions, which can be considered as a convenience sample due to the differences in the response rate across the eight institutions. The sample reported the following gender ratio: 78.7% female, 20.9% male and 0.4% other. Both undergraduate and graduate students participated in the survey and reported a mean age of 23.5 years (*SD* = 5.57), while the median value with regard to semester progress was four (2nd year of study). Over 70.7% were enrolled in a teacher education programme to become primary (25%) or secondary school teachers. Concerning their living situation during the restrictive measures taken to combat the pandemic, only 12.3% of participants indicated that they lived alone, while over 86.5% reported living with other people (median number of three people with a range between 1 and 12). All participants completed consent forms and agreed to anonymous data processing for scientific purposes.

### 4.2. Measures

#### 4.2.1. Basic Psychological Needs Satisfaction and Frustration (BPNSF)

The German Basic Psychological Need Satisfaction and Frustration Scale [6,45] was used to assess the satisfaction and frustration of the needs of autonomy, competence and relatedness in university students. A total of 24 items were rated on a 5-point Likert scale (1 = ‘does not apply at all’ to 5 = ‘applies completely’). Sample items and reliability coefficients are shown in Table 1.

The instrument achieved good to very good levels of scale reliability (see Table 1) and, as result of a *CFA* (*CFA* = Confirmatory Factor Analysis; *CFI* = Comparative Fit Index; *TFI* = Tucker-Lewis Index; *SRMR* = Standardized Root Mean Square Residual; *RMSEA* = Root Mean Square Error of Approximation), a good factor validity (χ^2^_(237)_ = 1566; *TLI* = 0.92; *CFI* = 0.93; *RMSEA* = 0.06; *SRMR* = 0.05). Based on a model comparison [46], we tested the factor validity of the six-factor-solution measuring all six dimensions separately, in comparison with a two-factor model with two aggregated factors for satisfaction and frustration. The two-factor model showed an unacceptable model fit (χ^2^_(251)_ = 6978; *TLI* = 0.63; *CFI* = 0.66; *RMSEA* = 0.11; *SRMR* = 0.12). Moreover, the six-factor-solution reported very high covariances between the frustration and satisfaction subscales of each BPN, with values between *r* = −0.76 and −0.92.

#### 4.2.2. Motivational Regulation

The motivational regulation of learning motivation was assessed using a validated German version [18] of an instrument partially based on the Academic Motivation Scale [25] and the Learning Self-Regulation Questionnaire [47]. The subscale ‘introjected-avoidance and approach’ was adapted from Sheldon and colleagues [23]. Minor adaptations to the wording of the items and the prompt specified the context, through reference to distance learning introduced during the pandemic restrictions.

A total of 15 items were rated on a 7-point Likert scale (1 = ‘does not apply at all’ to 7 = ‘applies completely’). One sample item for each subscale is shown, with its reliability coefficient, in Table 2. The *CFA* indicated a good factor validity of the instrument (χ^2^_(80)_ = 628; *TLI* = 0.94; *CFI* = 0.96; *RMSEA* = 0.06; *SRMR* = 0.05).

#### 4.2.3. Vitality in the Context of Learning

The German version of the Subjective Vitality Scale [27] was used to measure students’ vitality with seven items. The prompt specified the context by referring to the current situation of distance-learning. Participants answered prompts (e.g., ‘At this moment, I feel alive and vital’) on a 7-point Likert-type scale (1 = ‘not true’ to 7 = ‘very true’). The instrument achieved high levels of scale reliability (ω = 0.93) and the CFA showed good factor validity (χ^2^_(14)_ = 95.8; *TLI* = 0.99; *CFI* = 0.99; *RMSEA* = 0.06; *SRMR* = 0.02).

#### 4.2.4. Contextual Factors of Distance-Learning

Three independent variables were included to reflect the relevance of distance learning. First, the estimated ‘difference in the weekly workload’ was computed by subtracting the estimated number of study-related working hours before the move to online-based learning from the estimated number of study-related working hours after the change. Second, both ‘satisfaction with technology’ (specified in the prompt as satisfaction with technological equipment in regard to hardware, internet connection and additional IT equipment) and ‘perceived overload’ were indicated on a slide-bar with 100 units (1 = ‘very dissatisfied’ to 100 = ‘very satisfied with technical equipment’; 1 = ‘fully unchallenged’ to 100 = ‘fully overburdened with regard to distance learning’).

### 4.3. Study Procedure and Analysis

The goal of our study was to analyse the relationship between contextual factors, satisfaction and frustration of basic psychological needs, and motivational regulation towards subjective vitality in the context of university students. To determine the effect of the independent (contextual factors) and mediating variables (BPNSF and motivational regulation), we implemented a mediation model based on structural equation models (SEMs [48]). Additionally, we included age, gender and the semester as covariates, to control for confounding effects.

Besides chi-square statistics, we tested model validity by following fit measures (i.e., *TLI*, *CFI*, *RMSEA* and *SRMR*, [46]). Mediation effects were interpreted according to the typology of mediations presented by Zhao, Lynch and Chen [49]. For the statistical analyses, the package lavaan [50] for the statistical framework *R* [51] was adopted.

## 5. Results

Following an inspection of the bivariate correlations (see Table 3), we applied structural equational modelling (SEM) to investigate the relations between the variables of *BPNSFS* and vitality.

Due to the high collinearity between some scales of the *BPNSFS* (e.g., between satisfaction and frustration for the need of competence [*r* = −0.92, *p* < 0.001]), which resulted in misleading standardised beta weights larger than one [52], we computed two separate SEMs. Since an aggregated solution of the *BPNSFS* was not possible due to poor model fit, we opted to estimate two separate models, one for the satisfaction and one for the frustration of *BPN*. Both models reflected a good fit [46]: *SEM 1:* χ^2^_(642)_ = 2631.31, *TLI* = 0.93, *CFI* = 0.94, *RMSEA* = 0.04, *SRMR* = 0.05; *SEM 2:* χ^2^_(642)_ = 2696.92, *TLI* = 0.93, *CFI* = 0.94, *RMSEA* = 0.04, *SRMR* = 0.06.

**Figure 1:**
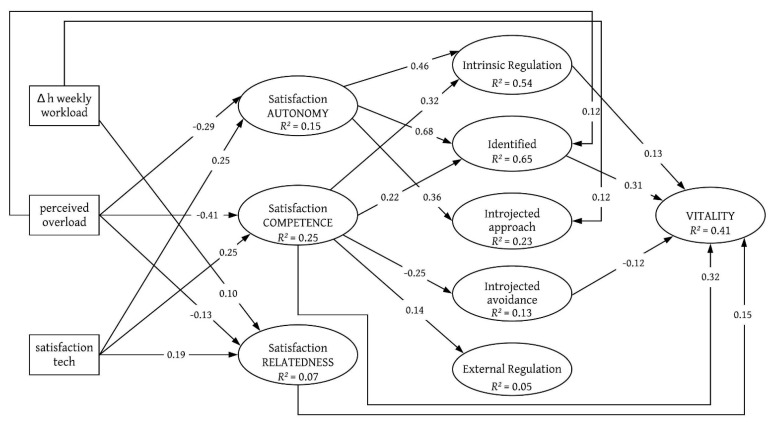
Structural Equation Model (SEM) indicating the standardised regression weights of the independent manifest variables, needs satisfaction and motivational regulation on subjective vitality, controlled for possible confounding effects by the variables of age, gender and study semester. All regressions and covariances were modelled, but only significant path weights (*p* < 0.01) are depicted, for visual clarity. Model fit: χ^2^_(642)_ = 2631.31, *TLI* = 0.93, *CFI* = 0.94, *RMSEA* = 0.04, *SRMR* = 0.05.

In SEM 1 (see Figure 1) we see that the perceived study context was associated both with basic psychological needs and the regulation of motivation. *Perceived overload* was associated with lower satisfaction for the needs of autonomy (β = −0.29, *p* < 0.001), competence (β = −0.41, *p* < 0.001) and relatedness (β = −0.13, *p* < 0.001), while an *increased workload* was linked to higher levels of relatedness (β = 0.10, *p* < 0.01). Unlike *perceived overload*, *satisfaction with technological equipment* was a positive predictor of all three needs (β = 0.25, *p* < 0.00 for both autonomy and competence; β = 0.19, *p* < 0.001 for relatedness). For motivational regulation, both the contextual factors and basic psychological needs reported significant paths: *intrinsic regulation* was associated with satisfaction of autonomy (β = 0.46, *p* < 0.001) and competence (β = 0.32, *p* < 0.001). *Identified regulation* reported positive regression coefficients with BPN satisfaction of autonomy (β = 0.68, *p* < 0.001) and competence (β = 0.22, *p* < 0.001) and, by a direct path, from *perceived overload* (β = 0.12, *p* < 0.001).

**Figure 2:**
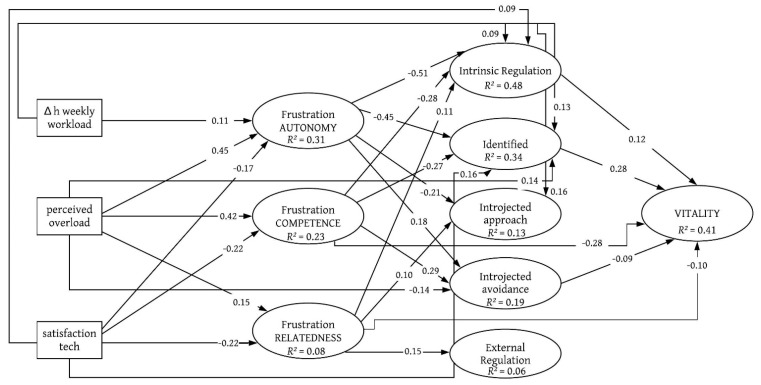
Structural Equation Model (SEM) indicating the standardised regression weights of the independent manifest variables, needs frustration and motivational regulation on subjective vitality, controlled for possible confounding effects by the variables of age, gender and study semester. All regressions and covariances were modelled, but only significant path weights (at least *p* < 0.01) are depicted for visual clarity. Model fit: χ^2^_(642)_ = 2696.92, *TLI* = 0.93, *CFI* = 0.94, *RMSEA* = 0.04, *SRMR* = 0.06.

For *introjected regulation*, the SEM reported a positive coefficient both with the satisfaction of autonomy (β = 0.36, *p* < 0.001) and *difference in workload* (β = 0.12, *p* < 0.001) on *introjected approach*, while the BPN satisfaction for competence reported a negative coefficient with *introjected avoidance*. BPN satisfaction for competence was also associated with higher levels of *external regulation* (β = 0.14, *p* < 0.01). Students’ vitality was linked to satisfaction in competence (β = 0.32, *p* < 0.001) and relatedness (β = 0.15, *p* < 0.001) and to motivational regulation, with positive paths for *intrinsic regulation* (β = 0.13, *p* < 0.001) and *identified regulation* (β = 0.31, *p* < 0.001), and a negative path coefficient for *introjected avoidance* (β = −0.12, *p* < 0.001). The results for the frustration of the BPN are depicted in SEM 2 (Figure 2). Autonomy frustration was significantly associated with all three independent variables. A *higher workload* (β = 0.11, *p* < 0.001) and *perceived overload* (β = 0.45, *p* < 0.001) during distance learning were associated with greater frustration of autonomy. However, *satisfaction with technological resources* is associated with lower frustration of autonomy (β = −0.17, *p* < 0.001). *Satisfaction with technology* was also associated with lower levels of frustration for competence and relatedness (both β = −0.22, *p* < 0.001). *Perceived overload* was linked to higher frustration of the BPN for competence (β = 0.42, *p* < 0.001) and for relatedness (β = 0.15, *p* < 0.001).

As can also be seen in SEM 2 (see Figure 2), the motivational regulation was linked both with contextual factors and basic psychological needs: *Intrinsic regulation* was associated negatively with BPN frustration of autonomy (β = −0.51, *p* < 0.001) and competence (β = −0.21, *p* < 0.001); the frustration of the BPN of relatedness was positively linked to *intrinsic motivation* (β = 0.11, *p* < 0.001). In addition, two significant distal paths were found for *satisfaction with technology* and *workload-difference*, with both showing a positive relationship towards *intrinsic regulation* (β = 0.09, *p* < 0.001).

For *identified regulation,* the model reported a significant regression path for the frustration of autonomy (β = −0.45, *p* < 0.001) and competence (β = −0.27, *p* < 0.001), as well as a direct path from *perceived overload* (β = 0.14, *p* < 0.001), *satisfaction with technology* (β = 0.09, *p* < 0.01) and *workload-difference* (β = 0.13, *p* < 0.001). The frustration of autonomy (β = −0.21, *p* < 0.001) and competence (β = −0.10, *p* < 0.01) were linked to lower levels of *introjected approach*, while *satisfaction with technology* (β = 0.10, *p* < 0.001), and *workload-difference* (β = 0.16, *p* < 0.001) were associated with higher levels. *Introjected-avoidance regulation* was positively linked to the frustration of autonomy (β = 0.18, *p* < 0.001) and competence (β = 0.29, *p* < 0.001), while for *perceived overload* the model reported a negative coefficient (β = −0.14, *p* < 0.001). *External regulation* was positively predicted by the frustration of the BPN for relatedness (β = 0.15, *p* < 0.001).

Vitality was associated with BPN frustration and motivational regulation: the frustration of competence (β = −0.28, *p* < 0.001) and relatedness (β = −0.10, *p* < 0.001) were associated with lower subjective vitality; *intrinsic regulation* (β = 0.12, *p* < 0.01) and *identified regulation* (β = 0.28, *p* < 0.001) reported positive regression paths, while *introjected-avoidance* reported a negative path coefficient (β = −0.09, *p* < 0.01) on students’ subjective vitality.

### 5.1. Mediation Analysis

We tested the interaction between basic psychological needs, motivational regulation and vitality for mediation effects, according to protocols promoted by Zhao and colleagues [49]. For both models, interaction with motivational regulation resulted in a full mediation of the effects of frustration and satisfaction of autonomy on vitality by *intrinsic* and *identified motivation*, confirming the non-significant direct path between BPN autonomy and vitality, the ‘indirect-only effect’ [49] (p. 201). Concerning the need for relatedness, neither model reported mediation through motivational regulation: here, both the regression weight of satisfaction (β_direct_ = 0.15, *p* < 0.001) and frustration (β_direct_ = −0.10, *p* < 0.001) of the BPN for relatedness was significant for the direct effect only. The BPN for competence was, however, associated with several mediating interactions with the regulation styles. In addition to the direct effect of the satisfaction of competence on vitality (β_direct_ = 0.32, *p* < 0.001), a complementary mediation by *intrinsic* (β_indirect_ = 0.04, *p* < 0.01; β_total_ = 0.36, *p* < 0.001), *identified* (β_indirect_ = 0.07, *p* < 0.001; β_total_ = 0.39, *p* < 0.001) and *introjected-avoidance regulation* (β_indirect_ = 0.03, *p* < 0.001; β_total_ = 0.35, *p* < 0.001) was reported. Frustration of the BPN for competence reported the same mediation effect in analogous modes. Besides the direct effect of the frustration of the need for competence on vitality (β_direct_ = −0.28, *p* < 0.001), a complementary mediation by *intrinsic* (β_indirect_ = 0.03, *p* < 0.01; β_total_ = −0.31, *p* < 0.001), *identified* (β_indirect_ = −0.07, *p* < 0.001; β_total_ = −0.35, *p* < 0.001) and *introjected-avoidance regulation* (β_indirect_ = 0.03, *p* < 0.001; β_total_ = 0.35, *p* < 0.001) was reported.

### 5.2. Outcome Variance

In both models, 41% of variance for the outcome variable of *vitality*, and up to 54% and 65% of outcomes concerning intrinsic and identified regulation, respectively, could be explained by the SEMs. The BPN for relatedness (*R*^2^ = 0.07/08) and external regulation (*R*^2^ = 0.05/06) reported only small effect sizes.

## 6. Discussion

The current study investigated whether students’ workload, perceived overload and satisfaction with their applied technological resources predict their basic needs’ satisfaction and frustration, respectively, as well as their motivational regulation in the enforced digital semester during the pandemic restrictions. Moreover, students’ needs satisfaction and frustration were each tested as predictors of their motivational regulation and as predictors of students’ individual vitality in that semester.

With regard to the students’ satisfaction with their technical equipment, our assumptions were confirmed (H1). Students’ satisfaction with technical resources was a predictor of the satisfaction of their basic needs; dissatisfaction with technical resources predicted BPN frustration. The more satisfied students were with their technical resources, the more able they were to satisfy their needs. Where there was a lack of technical resources or unsatisfactory resources, students could not work on tasks to an adequate standard. This may lead to an increase in task complexity, resulting in an imbalance between the requirements of the task and the ability of the students and, consequently, a low perception of competence [3]. Since the basic needs for competence and autonomy are mutually dependent [53], similar effects on students’ need for autonomy were assumed; these can be confirmed by the current data. The negative effects of unsatisfying technical resources on the need for autonomy are concordant with the results by Hartnett [38], who found technical restraints to be an autonomy-undermining influence. In line with our results, Lao and Gonzales [54] point out that adequate technical equipment is one key factor for effective online courses.

Rasheed [55] assumed organizational barriers to distance learning with regard to technological issues on all levels of education—students, teachers, staff, and faculty. Such barriers become evident when students only have limited experience with technology. Therefore, adequate technical support is needed, especially because this support has a crucial impact on students’ satisfaction with online courses [56]. Opportunities for technical support are outlined in Section 6.2.

As expected, perceived overload was not only a predictor of reduced satisfaction but also of frustration of the three basic needs (H1). Perceived overload indicates that the student’s abilities are not aligned with the challenges pending. This imbalance leads to the frustration of the need for competence [3]. Earlier findings suggest missing feedback as well as ambiguous instructions besides technical problems as sources of frustration in distance learning [57,58]. Missing feedback and ambiguous instructions represent an environment that is low in structure and can affect the students’ need for competence negatively [59]. Since the needs for autonomy and competence are interdependent [3,53], it is not surprising that the same correlations were found with the need for autonomy. Regarding students’ perceived satisfaction and frustration of the need for relatedness, it may be assumed that when students perceive overload, they limit their social contacts in order to counteract this overload. Moreover, students’ relatedness to peers, as well as lecturers and supervisors, was limited due to the pandemic restrictions [57].

High demands such as perceived overload that was shown to affect the students’ needs negatively can ultimately result in students’ burnout [60]. Our results suggest that in order to counteract overload and subsequently burnout, need-satisfying measures should be implemented, which are presented in Section 6.2.

Interestingly, the estimated difference in weekly workload before and during the pandemic restrictions yielded inconsistent findings. Here, the presumed relationships with basic needs cannot be confirmed (H1). The only outcomes that can be accurately predicted by workload are the frustration of the need for autonomy, and satisfaction of the need for relatedness. This finding is consistent with Hartnett [38], who identified workload as a crucial autonomy-undermining influence: the greater the workload, the more the need for autonomy is restricted. Contrary to the assumption of the reverse conclusion, our findings do not reflect a decreased satisfaction of the basic need for autonomy caused by increased workload [5,53]. With regard to the small regression coefficient on relatedness, it could be assumed that a high workload in online study requires students to work more closely with each other and with the lecturer; students are more aware of the fulfilment of this need since opportunities for interaction are more limited in comparison to their regular studies [2,38]. In line with our assumptions, Kember [61] suggests that students strive more actively for interactions when having a high workload. Moreover, he found evidence that relationships between the students can reduce the perception of a high workload. However, contrary to our results regarding relatedness, Basson and Rothmann [62] showed that workload has a negative impact on the satisfaction of the need for relatedness.

When compared to the effects of perceived overload, the impact of the students’ workload appears minimal, suggesting that the perception of overload has a stronger effect on students’ basic needs satisfaction and frustration than the estimated weekly workload difference. Such inconsistencies might be attributed to differences between the students’ perception of their workload and their actual workload, which will be addressed Section 6.1.

In the satisfaction model, the basic needs for autonomy and competence show positive effects on intrinsic and identified regulation (cf. H2; [5]). In addition, the need for autonomy shows a positive effect on introjected approach regulation, which indicates the autonomous character of this regulation type, providing empirical evidence that it is necessary to consider introjected regulation as differentiated [63]. These findings are in line with the second hypothesis, with the exception of relatedness, which had no effect on any regulation in the satisfaction model. Moreover, frustration of the needs for autonomy and competence negatively predicted students’ autonomous motivational regulation (intrinsic, identified and introjected approach; [5]). Autonomy and competence frustration both positively predicted external regulation [15], while frustration of the need for relatedness positively predicted students’ external regulation to a minor extent.

Taking both models into account, these results suggest that the basic needs for competence and autonomy are particularly important for students’ self-determined motivational regulation under restrictions such as those introduced during the pandemic, whereas the need for relatedness seems to play a subordinate role. It should be noted that negative correlations have been recorded between the frustration of the need for relatedness and the external regulation variables, indicating that suppression effects may be present here [52]. Moreover, our results suggest that introjected regulation needs to be considered in a differentiated way. Introjected approach regulation seems to be more self-determined, whereas introjected avoidance seems to be more controlled, in line with the findings of [63]. Neither the satisfaction nor the frustration of the three basic needs, however, had an effect on introjected avoidance regulation. With regard to external regulation, frustration of the students’ needs for autonomy and competence had a positive impact. This agrees with our assumptions (H2) and the findings of [64].

Regarding the third hypothesis, our data reveal that the needs for competence and relatedness had a positive effect on subjective vitality, whereas a negative effect is found in the frustration model [29,31,43]. These results suggest that satisfying the needs for competence and relatedness is more vital in extreme situations, whereas the option to act autonomously is of minor relevance in such circumstances. It seems to be more important for students to feel competent in these new, challenging tasks, as well as to have close relationships with others in insecure times and to combat the crisis together, than to have the opportunity to choose, act voluntarily and originate their own behaviour. At university level, Miksza, Evans and McPherson [65] found evidence that the quality of peer-relationships, while a positive predictor of vitality, does not moderate the effect of stress on vitality. These findings highlight the importance of relatedness that already existed prior to the pandemic restrictions. Contrary to our results, Ryan and Deci [11] point out that vitality arises from the satisfaction of all three needs. Moreover, they assume that the type of regulation has an impact on an individual’s vitality.

Ryan and Deci [3] suggest that when actions are influenced by external factors, such as control or pressure, vitality may suffer. Motivational regulation and, more specifically, autonomous forms of motivational regulation, affect vitality positively [66,67], whereas controlled forms of motivational regulation show negative effects [66]. In line with previous findings and our third hypothesis, self-determined motivational regulations (intrinsic and identified) positively predicted vitality in the satisfaction and frustration model. Moreover, introjected avoidance, a controlled form of regulation, negatively predicted subjective vitality in both models [23,43].

However, the mediating effect of motivational regulation on the relationship between vitality and the satisfaction or the frustration of basic needs must be considered when discussing the importance of each need. As expected, and in line with McDonough and Crocker [44], we found such mediating effects for the needs for competence and autonomy, but not for the need for relatedness. Similar effects of the needs on vitality were found in the satisfaction and frustration model.

Autonomy showed full mediation through the motivational regulation types [44], suggesting that autonomy has an impact on vitality, despite no direct effect shown in the SEM models. In comparison, the need for competence showed a complementary mediation—mediation through the regulation types as well as a direct effect on vitality [44]. The mediating effects were found for the three types of motivational regulation (introjected avoidance, identified and intrinsic), reflecting both a positive and negative relationship between the BPN for competence and these regulation types. Since our data show direct and indirect effects of the need for competence on vitality, the need for competence seems to play a particular role in distance learning and managing new digital and technical resources. The small direct effect, together with the lack of a mediating effect of motivational regulation, suggests that relatedness was of minor importance for student vitality during the semester of online teaching. This result is contrary to the findings of McDonough and Crocker [44] and may be attributed to the particular circumstances of online learning. Students seem to function effectively when learning online, and are well aligned with their inner selves when they see themselves as autonomous and competent [29,30]. Unexpectedly, feelings of relatedness with peers and lecturers seem to have no impact on their functioning or energy reserves.

### 6.1. Practical Implications

In line with SDT, need satisfaction and frustration have emerged as significant variables in students’ motivational regulation and vitality in our study. The detailed analysis of these relations not only affirms these expected relations in online learning settings but also allows the deviation of practical implications for designing and accompanying distance learning settings. The experience of psychological needs satisfaction is context-sensitive and lecturers can actively contribute to support students’ needs for autonomy, competence and relatedness. For example, strategies to promote relatedness include showing respect, appreciation and empathy as well as providing (emotional) support in difficult times [68]. Moreover, collaborative tasks allowing students to work together can be implemented. Various structuring measures can be used to promote the need for competence [59,68]. These measures include communicating clear expectations and goals, offering a schedule for students to organize their learning, informative feedback and opportunities for self-evaluation or peer-evaluation [59]. Providing choices from which students can select assignments and topics according to their interests, demonstrating personal significance of assignments and actions in online sessions, and addressing and respecting negative feelings are described as measures to foster the need for autonomy [68]. If students perceive their needs as satisfied by these measures, they may benefit in terms of their motivational regulation and vitality in their online studies, as our findings indicate. However, supporting psychological needs satisfaction is not sufficient. In addition, lecturers have to be aware of needs frustration. Previous research indicates that lecturers can act as autonomy-supportive and pressurizing at the same time—often without being aware of it [69]. In environments that aim to prevent needs frustration, it is important to avoid social comparisons and competition between the students and to abstain from external forces such as rewards, unclear deadlines or threats [68]. The COVID-19 pandemic proves to be challenging for students and lecturers but a need-sensitive approach to online learning and teaching can be realized with manageable effort and can ease the pressure for both students and lecturers [70].

Our results suggest that students need satisfying technical equipment to work autonomously and perceive themselves as competent and related in their online studies. Although university instructors hardly have an impact on the technical equipment of their students, measures can be taken which, for example, aim at a competent handling of the technical requirements. In addition, universities could offer students a pool of technical equipment that is only needed temporarily or lecturers can adjust their assignments by allowing differentiated methods or variable time spans [71]. Finally, university instructors should not only focus on the workload, but also take into account the subjective perception of the students’ workload. With the assessment of the subjective perception, perceived under- or overload can be identified, and competence-supportive measures to counteract this under- or overload, which have just been described, can be implemented accordingly. In addition, it should be considered that the workload measured in our study is a self-report and not an objective measure. In future studies, for example, digital tools could be used to record the actual learning time.

To sum up, in online learning arrangements regular online exchange with students, e.g., in so-called tech consultation hours or feedback sessions concerning content and methods, can help to reduce the risk of overload and to align work tasks with the technical and individual resources of students. Considering the extraordinary circumstances caused by the pandemic, it seems worthwhile to pay special attention to the well-being of students studying in distance settings and the influential role of lecturers and learning arrangements in this respect [72].

Despite these promising results for the semester of online learning during the pandemic, some limitations of our study need to be addressed. These limitations are discussed in the following chapter.

### 6.2. Limitations

First, the variables were collected cross-sectionally and simultaneously; therefore, we are not able to investigate causal effects but only relationships. Thus, future studies using longitudinal data to support our findings would be valuable. Secondly, our sample comprises students from different disciplines (e.g., teacher training courses and subject-specific studies) and from different countries, which allowed for a more representative picture of the situation in Central European institutions for higher education. In a preliminary analysis, measurement invariance was confirmed for the study variables. Further analysis could additionally implement group comparisons to determine whether group-specific interactions occur in the SEM. Thirdly, we used only self-reporting measures. Self-reports on issues such as student workload may lack predictive validity [73]. However, by asking students to estimate their working hours before and during the lockdown as two separate items, rather than directly guessing the workload difference, we aimed to achieve a more accurate approach to estimating the workload difference by computing the subtraction in the analysis. In the design of future studies, the application of additional objective measures for validating self-reported information could be considered.

Nevertheless, two studies need to be cited regarding self-report measures in the context of workload—Kember [61] as well as Kember and Leung [74] state that actual workload is, in fact, very difficult to measure as it consists of a complex composition of the contact time in a course, the time to complete the assignments of the course and time required to understand the contents of the course. The associated workload with regard to the assignments and understanding the content might differ between students because they may employ approaches to learning in a course that vary in the depth of processing. Kember and Leung [74] further hypothesise that the actual workload is only weakly related to the perceived workload. Moreover, Kember [61] found evidence that the perception of workload is a more important predictor for workload than the actual workload represented by the contact hours in the course or time spend studying for the course.

Furthermore, the limitations of the cross-sectional design could be reduced by implementing a diary-based approach. Fourthly, the items of the introjected avoidance scale have not yet been validated; data collected with this scale should therefore be treated with caution. However, the current findings on the relationship between basic needs and motivational regulation are consistent with previous findings and assumptions [5,6,45]. Currently, a validation of the items is in progress.

## 7. Conclusions

The study stresses the importance of an adequate technical equipment and workload for students during the COVID-19 pandemic to satisfy their satisfaction of needs and to support their vitality. In times of severe restrictions and consequential new forms of learning and teaching, this seems to be especially true for the needs for competence and autonomy, while the need for relatedness seems to play a minor role. To reduce perceived overload and to ensure that work tasks correspond to students’ competence and technical resources, lecturers might reconsider their teaching style, adjust the design of the (online) learning environment, and offer additional support. Key need-supportive strategies, such as transparent communication, responsiveness, acting understanding and supportive, and time for collaboration between students and with lecturers can be beneficial for students during these difficult times. The pandemic situation is not only a challenge for students but for lecturers as well. Although lecturers themselves have to cope with additional challenges during online teaching, considering the psychological needs of their students can reciprocally contribute to maintain their motivation for teaching [70].

## Figures and Tables

**Table 1:** Items and Scale Reliabilities of the BPNSF.

Subscales	Scale Reliability ω	Sample Item
Satisfaction		
Autonomy	0.76	I feel that my decisions regarding my studies reflect what I really want.
Competence	0.90	I feel I can successfully complete difficult tasks for my study program.
Relatedness	0.78	I feel close and connected with other people from university who are important to me.
Frustration		
Autonomy	0.85	In my studies, I feel pressured to do too many things.
Competence	0.84	At university, I feel like a failure because of the mistakes I make.
Relatedness	0.72	I feel that people from university who are important to me are cold and distant towards me.

**Table 2:** Items and Scale Reliabilities of the Motivational Self-Regulation.

Regulation Subscales	Scale Reliability ω	Sample Item
Intrinsic	0.91	I really enjoy studying and working for university.
Identified	0.75	Mainly, I study to be more proficient and to develop myself further.
Introjected Approach	0.81	I study because I want to prove to myself that I am capable of successfully completing this ‘distance learning-semester’.
Introjected Avoidance	0.84	I study for this online-program because otherwise I would feel guilty.
External	0.70	I study mainly because I want to obtain an academic degree.

**Table 3:** Means, Standard Deviation, and Correlations of the Measures.

	M	SD	1.	2.	3.	4.	5.	6.	7.	8.	9.	10.	11.
1. Δ Workload Hours	7.88	12.5	—										
2. Perceived Overload	68.7	17.3	0.45 ***	—									
3. Satisfaction Tech	72.9	26.9	−0.04	−0.06 *	—								
4. BPN Sat. Autonomy	3.14	0.86	−0.11 ***	−0.26 ***	0.24 ***	—							
5. BPN Sat. Competence	3.28	0.98	−0.16 ***	−0.39 ***	0.25 ***	0.57 ***	—						
6. BPN Sat. Relatedness	3.07	0.94	0.05 *	−0.06 *	0.17 ***	0.32 ***	0.35 ***	—					
7. BPN Fru. Autonomy	3.60	1.00	0.29 ***	0.45 ***	−0.18 ***	−0.63 ***	−0.51 ***	−0.18 ***	—				
8. BPN Fru. Competence	2.32	1.04	0.12 ***	0.37 ***	−0.21 ***	−0.45 ***	−0.79 ***	−0.30 ***	0.46 ***	—			
9. BPN Fru. Relatedness	1.99	0.81	0.03	0.10 ***	−0.19 ***	−0.25 ***	−0.34 ***	−0.58 ***	0.24 ***	0.44 ***	—		
10. Intrinsic R.	3.32	1.70	−0.08 ***	−0.28 ***	0.22 ***	0.61 ***	0.59 ***	0.23 ***	−0.56 ***	−0.45 ***	−0.17 ***	—	
11. Identified R.	4.83	1.36	0.02	−0.10 ***	0.19 ***	0.57 ***	0.49 ***	0.23 ***	−0.39 ***	−0.35 ***	−0.15 ***	0.057 ***	—
12. R. Introjected Approach	4.30	1.57	0.10 ***	−0.01	0.12 ***	0.30 ***	0.22 ***	0.13 ***	−0.14 ***	−0.09 ***	−0.01	0.34 ***	0.50 ***
13. R. Introjected Avoidance	3.73	1.67	0.05	0.07 **	−0.08 **	−0.18 ***	−0.25 ***	−0.09 ***	0.25 ***	0.33 ***	0.19 ***	−0.18 ***	−0.04
14. External R.	4.51	1.47	0.03	0.02	−0.02	−0.01	0.03	−0.03	0.07 **	0.04	0.10 ***	0.06 *	0.06 **
15. Vitality	4.18	1.36	−0.13 ***	−0.26 ***	0.22 ***	0.42 ***	0.55 ***	0.29 ***	−0.42 ***	−0.51 ***	−0.30 ***	0.48 ***	0.40 ***

Note. * *p* < 0.05, ** *p* < 0.01, *** *p* < 0.001; Abbreviations: M = Mean; SD = Standard Deviation; BPN = Basic Psychological Need; Fru. = Frustration; Sat. = Satisfaction; R. = Regulation.

## Data Availability

The datasets generated during and analysed in the current study are not publicly available due to further, ongoing research projects but are available from the corresponding author on reasonable request.
